# Correction: A trustworthy hybrid model for transparent software defect prediction: SPAM-XAI

**DOI:** 10.1371/journal.pone.0343580

**Published:** 2026-02-24

**Authors:** Mohd Mustaqeem, Suhel Mustajab, Mahfooz Alam, Fathe Jeribi, Shadab Alam, Mohammed Shuaib

The citations in Table 12 [1] are incorrect. Additionally, the articles cited as References 59 and 60 were erroneously included and should not be cited in the article. An updated Table 12 is provided with correct citations.

There is an error in the ninth sentence of the second paragraph of the Introduction section; the technique used in Reference 5 is erroneously referred to as “Microtext-Deep Neural Networks”. The correct technique is “Multi-Task Deep Neural Networks”.

The authors apologize for the errors in the published article.

**Table 12:** Analysis of previously developed models with SPAM-XAI model.

Dataset	Models	Precision	Recall	F-m	Accuracy	AU-ROC
CM1	Naïve Bayes [2]	86.20	78.65	34.09	64.57	70.0
	Random Forest [2]	71.10	71.29	32.17	60.98	76.0
	C4.5 Miner [45]	74.91	74.66	27.68	66.71	53.0
	Immunos [45]	73.65	75.02	30.99	66.03	63.0
	ANN-ABC [45]	75.00	81.00	33.00	68.00	77.0
	HSOM [45]	70.12	78.96	30.65	72.37	80.0
	SVM [45]	81.20	79.08	31.27	78.69	50.0
	Majority vote [45]	79.80	80.00	30.46	77.01	81.0
	AntMiner+ [45]	80.65	78.88	30.90	73.43	84.0
	ADBBO-RBFNN [45]	81.92	80.96	29.71	82.57	90.0
	NN GAPO + B [2]	–	–	–	74.40	–
	DT [2]	83.30	74.23	81.20	73.49	37.0
	KNN [2]	83.90	84.70	84.30	–	47.0
	MLP [2]	90.40	95.50	92.90	86.73	63.4
	HybridModel (PC-SVM) [2]	96.10	99.00	97.00	95.20	–
	Hybrid approach (GA-DNN) [2]	80.32	97.32	89.09	97.50	–
	GWOFS-MLP Model [2]	98.27	86.36	91.93	92.72	81.60
	**SPAM-XAI model**	**95.00**	**96.00**	**95.00**	**98.20**	**91.0**
PC1	Naïve Bayes [2]	94.70	87.98	97.20	60.00	58.79
	Random Forest [2]	95.00	82.31	96.00	63.98	85.00
	C4.5 Miner [45]	76.58	81.76	34.05	62.18	68.0
	Immunos [45]	81.99	79.66	36.92	61.73	64.0
	ANN-ABC [45]	89.00	83.00	33.00	65.00	–
	HSOM [45]	86.79	85.67	35.67	95.87	–
	SVM [45]	80.98	86.59	98.00	92.45	50.0
	Majority vote [45]	84.61	84.37	30.98	92.50	–
	AntMiner+ [45]	89.34	87.12	26.11	91.85	–
	ADBBO-RBFNN [45]	90.89	89.33	20.24	–	–
	DT [2]	93.00	99.00	96.00	92.87	65.61
	KNN [2]	–	–	28.60	94.90	82.90
	MLP [2]	–	–	46.20	96.56	77.90
	DNN [2]	94.00	99.00	97.00	93.00	–
	GWOFS-MLP Model [2]	99.24	95.65	97.40	95.03	84.00
	**SPAM-XAI model**	**97.01**	**99.00**	**98.00**	**97.51**	**79.0**
