# Supplementary material for: A trustworthy hybrid model for transparent software defect prediction: SPAM-XAI
Source: PLoS One. 2024 Jul 11;19(7):e0307112. doi: 10.1371/journal.pone.0307112 (PMC11239108; doi:10.1371/journal.pone.0307112)
Supplement: Attachment — Submitted filename: review comments.docx [file pone.0307112.s001.docx]

1. The paper lacks an introduction about software defects. It is necessary to include a section that defines software defects and discusses their nature and types to provide context and enhance the reader's understanding of the issues addressed.

2.Figures 2 and 3 are incomplete. Please ensure that all figures are fully developed and include all necessary labels, legends, and descriptions to effectively convey the intended information.

3.Equations 7, 8, 12 and 17 are incomplete. Please provide the full equations with all necessary terms and explanations to ensure clarity and accuracy.

4.Certain terms in Equation 17 are not defined in the paper. Please include the missing equations and provide clear definitions and explanations for each term to ensure the equation is fully understandable.

equation is missing

5.The paper's title mentions that the algorithm is transparent; however, this justification is not clearly provided in the text. Please elaborate on how and why the algorithm is considered transparent, including details on its design, implementation, and the ways it allows for easy understanding and verification by others.

6.For Tables 1, 2, and 3, please ensure that parameterized comparisons are included to facilitate a more thorough analysis. Additionally, any multi-line text should be moved to the supporting sections to maintain the clarity and conciseness of the tables

7.The validation of the algorithm should be conducted in real-time scenarios rather than solely relying on a portion of the dataset. Real-time validation will provide a more accurate assessment of the algorithm's performance in practical use cases and enhance its credibility and applicability.

8. Please specify which stage of the SDLC the algorithm is incorporated into. Elaborate on how integrating the algorithm at this stage impacts the overall SDLC process, considering a real-time scenario. Providing insights into how the algorithm affects requirements gathering, design, implementation, testing, or maintenance phases will enhance the understanding of its integration within the SDLC framework.
