# Supplementary material for: A trustworthy hybrid model for transparent software defect prediction: SPAM-XAI
Source: PLoS One. 2024 Jul 11;19(7):e0307112. doi: 10.1371/journal.pone.0307112 (PMC11239108; doi:10.1371/journal.pone.0307112)
Supplement: Attachment — Submitted filename: PONE-D-24-11437-Response-File..docx [file pone.0307112.s002.docx]

**Responses to Review Comments**

**Manuscript title:** A Trustworthy Hybrid Model for Transparent Software Defect Prediction: SPAM-XAI

**Manuscript ID: PONE-D-24-11437.R1**

We have incorporated the necessary changes and suggestions from the editor and reviewers in the revised version.

**Editor Comments**

**The paper promises to be about XAI for SPAM detection. However, there are not interpretation of reasons behind prediction of results? So what is eXplainable here. All methods are like KNN NB etc which are common ML algorithms.**

**Response:** Thank you for your insightful feedback. We apologize for any confusion caused by the name of our model, SPAM-XAI, and we appreciate the opportunity to clarify our work. We would like to clarify that the primary focus of this paper is on Software Defect Prediction (SDP), not SPAM detection. The name SPAM-XAI is derived from the techniques used within the model and is not related to SPAM detection.

Our SPAM-XAI model incorporates eXplainable AI techniques to provide transparency and interpretability in predicting software defects. The detailed explanations and visualizations of these contributions are provided on (pages 36 and 38), specifically in sections 7, 7.1, and 7.2.

1) **Data Imbalancing is the issue which is shared as research gap. however, nature of SPAM is about imbalance class. So, it is an imbalanced data problem inherently. obviously if i receive 1000 emails on gmail, only few will be SPAM so it has to be imblanced like MEDICAL diagnositc etc. so why to balance it?**

**Response:** Thank you for your comment regarding data imbalance and its relevance to our research. We appreciate the opportunity to clarify this aspect of our study.

We understand the inherent nature of data imbalance in certain domains, such as SPAM detection and medical diagnostics. However, it is important to note that our research specifically focuses on software defect prediction (SDP), not SPAM detection. We apologize for any confusion caused by the mention of the SPAM-XAI model in our paper, and we want to clarify that SPAM-XAI is designed for SDP.

In SDP’s context, we are dealing with highly imbalanced datasets. The dataset we use for our study is the NASA Metrics Data Program (MDP) dataset, which is well-known for its imbalance between defect and non-defect instances. We provide detailed information about the imbalance in Section 4.1.1 and on page no. 14 of the manuscript.

2) **PseudoCode is very poorly presented, better remove it, framework is good enough.**

**Response**: Thank you for your feedback regarding the presentation of the PseudoCode in our paper. We appreciate your input and acknowledge the need for improvement in this aspect.

3) **XAI is added in framework but it does not show how it AI has become XAI?**

**Response:** Thank you for your comment regarding the incorporation of eXplainable AI (XAI) techniques in our framework. We appreciate the opportunity to clarify this aspect of our work.

We acknowledge your observation regarding the need for clarity on how traditional AI techniques have been transformed into eXplainable AI (XAI) in our framework. Your feedback is valuable, and we aim to address this concern effectively.

In our paper, we have utilized the LIME (Local Interpretable Model-agnostic Explanations) technique to enhance the transparency of our predictive model. This technique is discussed in detail on page 20, specifically in Section 4.2.4 of the manuscript.

4) **no need of sharing what is confusion matrix**

**Response:** Thank you for your feedback. We will remove the section explaining the confusion matrix from the paper.

5) **equation numbers are missing and many other basic format issues.**

**Response:** Thank you for highlighting our manuscript’s formatting issues, including missing equation numbers. We appreciate your feedback and acknowledge the importance of addressing these issues.

**Reviewer #1**

1. **Revise Reference Numbering**

**Response:** Thank you for your comment. We have thoroughly reviewed the manuscript and made the necessary revisions to ensure the reference numbering is accurate and consistent.

2. **In the Introduction part, strong points of this proposed method should be further stated and organization of this whole paper is supposed to be provided in the end.**

**Response:** we appreciate your feedback regarding the Introduction section 1 and have made the necessary updates to address your suggestions from Page no. 3 to 5. Thank you for bringing these points to our attention, and we believe that these revisions will improve the overall quality of our manuscript.

3. **The Introduction is not enough.**

**Response:** We appreciate your feedback regarding the Introduction section. We have revised and expanded it to provide more comprehensive background information, motivation for the study, and a clear statement of the research objectives. We will ensure that the Introduction effectively sets the stage for the rest of the manuscript and outlines the significance of our research in the broader context of the field.

4. **The articles listed in References are old. (Most of them are out of date. For example, the papers published within 2 years are used to calculate CiteScore in SCOPUS.) The authors should survey past studies in detail. Besides, the authors should justify the effectiveness of the proposed method by comparing with state-of-the-art methods.**

**Response:** Thank you for your feedback. In response to your feedback, we have updated the references cited in our manuscript to include more recent articles. We have conducted a thorough survey of past studies to ensure that our literature review is comprehensive and up-to-date.

5. **The authors should compare the proposed method with other researchers’ methods. There are many previous works. The authors should emphasize the difference with other researchers’ methods. Add more comparison data.**

**Response:** Thank you for the comment. We have added in section 6.2 “SPAM-XAI Model Comparison with Prior State-of-the-Art” on page no. 32 and 33 of the manuscript.

6. **The problem definition of this work is not clear. In Sect. 2, the drawbacks of each technique should be described one by one. The authors should emphasize the difference with other methods to clarify the position of this work further.**

**Response:** Thank you for your feedback. We have made efforts to clearly state the problem definition in the Introduction section of our manuscript, specifically on page 3.

In response to the description of drawbacks, Section 2, titled “Literature Review,” describes the drawbacks of various techniques and previously developed models. Specifically, we have addressed the drawbacks of techniques in dealing with Section 2.2, “imbalanced class problem”, from pages 8 to 10.

Additionally, in Section 3, titled “Comparative Analysis of Various Algorithms with the SPAM-XAI Model,” we have further analyzed the drawbacks of different algorithms, providing a comparative analysis on pages 11 and 12.

We believe that these revisions enhance the clarity and comprehensiveness of our research.

7. **Figures are not clear**

**Response:** Thank you for bringing this to our attention, and we apologize for any inconvenience caused by the unclear figures. We have increased the resolution to 300 dpi, which should improve the quality of the figures and make them easier to interpret.

8. **Revise all abbreviations**

**Response:** Thank you for bringing this to our attention, and we apologize for any confusion caused by unclear or inconsistent abbreviations. We have carefully reviewed and revised all abbreviations used throughout the manuscript

9. **Analysis of complexity of the algorithm both theoretically and empirically must be added (Space and Time Complexity)**

**Response:** Thank you for the feedback. In response to your feedback, we have added a new section titled “Time Complexity and Computational Cost” in our manuscript on pages 35 and 36.

**Reviewer #2**

1. **The paper lacks an introduction about software defects. It is necessary to include a section that defines software defects and discusses their nature and types to provide context and enhance the reader’s understanding of the issues addressed.**

**Response:** Thank you for bringing this to our attention, We have added a new section 2.1 “Software Defects” on pages 6 and 7.

2. **Figures 2 and 3 are incomplete. Please ensure that all figures are fully developed and include all necessary labels, legends, and descriptions to effectively convey the intended information.**

**Response:** We appreciate your comment. In response to your feedback, we have reviewed and corrected Figures 2 and 3 in our manuscript.

3. **Equations 7, 8, 12 and 17 are incomplete. Please provide the full equations with all necessary terms and explanations to ensure clarity and accuracy.**

**Response:** Thank you for bringing this to our attention, and we apologize for any confusion caused by incomplete equations. In response to your feedback, we have completed Equations 7, 8, 12, and 17 on page no. 18, 19, 20 and 21.

4. **Certain terms in Equation 17 are not defined in the paper. Please include the missing equations and provide clear definitions and explanations for each term to ensure the equation is fully understandable.**

**Response:** Thank you for bringing this to our attention, and we apologize for any confusion caused by missing terms in Equation 17. We have included the missing terms in Equation 17 on page no. 21.

5. **The paper’s title mentions that the algorithm is transparent; however, this justification is not clearly provided in the text. Please elaborate on how and why the algorithm is considered transparent, including details on its design, implementation, and the ways it allows for easy understanding and verification by others.**

**Response:** Thank you for your feedback regarding the transparency of our algorithm. Our algorithm, named SPAM-XAI, incorporates several features designed to enhance transparency. We have utilized explainable AI (XAI) techniques, such as LIME, to provide insights into the model’s decision-making process. These techniques allow us to identify which features contribute most to the model’s predictions, facilitating easy understanding and interpretation.

In section 5. “Implementation” on page 25, we have provided detailed descriptions of the design and implementation of our algorithm.

6. **For Tables 1, 2, and 3, please ensure that parameterized comparisons are included to facilitate a more thorough analysis. Additionally, any multi-line text should be moved to the supporting sections to maintain the clarity and conciseness of the tables**

**Response:** Thank you for your valuable feedback. We have incorporated parameterized comparisons for Tables 1, 2, and 3 in section 3, “Comparative analysis of various algorithms with the SPAM-XAI model,” on pages 11 and 12. Additionally, we have moved any multi-line text to the supporting sections to maintain the clarity and conciseness of the tables.

7. **The validation of the algorithm should be conducted in real-time scenarios rather than solely relying on a portion of the dataset. Real-time validation will provide a more accurate assessment of the algorithm’s performance in practical use cases and enhance its credibility and applicability.**

**Response:** Thank you for your concern. We acknowledge the importance of real-time validation for assessing the algorithm’s performance in practical use cases. We plan to incorporate real-time validation in future work to enhance the credibility and applicability of the SPAM-XAI model.

8. **Please specify which stage of the SDLC the algorithm is incorporated into. Elaborate on how integrating the algorithm at this stage impacts the overall SDLC process, considering a real-time scenario. Providing insights into how the algorithm affects requirements gathering, design, implementation, testing, or maintenance phases will enhance the understanding of its integration within the SDLC framework.**

**Response:** Thank you for your comment. We have clarified the algorithm's integration stage in the SDLC on page 4 of the “Introduction” section. In real-time scenarios, we plan to incorporate detailed insights on how the algorithm affects various SDLC phases, including requirements gathering, design, implementation, testing, and maintenance, in our future work. This will enhance our understanding of its integration within the SDLC framework.
