# Supplementary material for: A trustworthy hybrid model for transparent software defect prediction: SPAM-XAI
Source: PLoS One. 2024 Jul 11;19(7):e0307112. doi: 10.1371/journal.pone.0307112 (PMC11239108; doi:10.1371/journal.pone.0307112)
Supplement: Attachment — Submitted filename: Response-FileR2-PONE-D-24-11437.docx [file pone.0307112.s003.docx]

**Manuscript title:** **A Trustworthy Hybrid Model for Transparent Software Defect Prediction: SPAM-XAI**

**Manuscript ID: PONE-D-24-11437.R2**

We have incorporated the necessary changes and suggestions from the editor in the revised manuscript.

**Journal Requirements:**
1) Please review your reference list to ensure that it is complete and correct. If you have cited papers that have been retracted, please include the rationale for doing so in the manuscript text, or remove these references and replace them with relevant current references. Any changes to the reference list should be mentioned in the rebuttal letter that accompanies your revised manuscript. If you need to cite a retracted article, indicate the article’s retracted status in the References list and also include a citation and full reference for the retraction notice.

**Response:** Thank you for your comment regarding the references. We have reviewed and updated the reference list according to the journal’s citation style, “Vancouver”. Specifically, we have made the following updates in references as per editorial suggestion:

- **Reference [34]:** Abdu A, Zhai Z, Abdo HA, Algabri R. Software Defect Prediction Based on Deep Representation Learning of Source Code From Contextual Syntax and Semantic Graph. IEEE Transactions on Reliability. 2024 Feb 1;73(2):820-34 <https://doi.org/10.1109/TR.2024.3354965>
- **Reference [55]:** Jiarpakdee J, Tantithamthavorn CK, Dam HK, Grundy J. An empirical study of model-agnostic techniques for defect prediction models. IEEE Transactions on Software Engineering. 2020 Mar 23;48(1):166-85. <https://doi.org/10.1109/TSE.2020.2982385>

**We have also checked for retracted articles and ensured that none are cited in our manuscript. Thank you for your guidance on this matter.**

**Regards**

Fathe Jeribi

[fjeribi@jazanu.edu.sa](mailto:fjeribi@jazanu.edu.sa)

On behalf of all authors
